# Coeliac Disease With Rheumatoid Arthritis: An Unusual Association

**DOI:** 10.14740/gr641w

**Published:** 2015-02-14

**Authors:** Synrang Batngen Warjri, Tony Ete, Taso Beyong, Bhupen Barman, Kyrshanlang G. Lynrah, Hage Nobin, Obang Perme

**Affiliations:** a Department of General Medicine, North Eastern Indira Gandhi Regional Institute of Health and Medical Sciences, Shillong, Meghalaya, India; b Department of Pathology, North Eastern Indira Gandhi Regional Institute of Health and Medical Sciences, Shillong, Meghalaya, India

**Keywords:** Coeliac disease, Autoimmune disease, Rheumatoid arthritis

## Abstract

Coeliac disease has a significant association with many autoimmune disorders. It shares many common genetic and immunological features with other autoimmune diseases. Gluten, a gut-derived antigen, is the driver of the autoimmunity seen in coeliac disease. The altered intestinal permeability found in coeliac patients, coupled with a genetic predisposition and altered immunological response, may result in a systemic immune response that is directed against sites other than the gut. Gut-derived antigens may have a role in the pathogenesis of other autoimmune disorders including rheumatoid arthritis. Here we report a case of adult coeliac disease associated with rheumatoid arthritis.

## Introduction

Coeliac disease is an autoimmune disease characterized by chronic intestinal inflammation in response to exposure to gluten, a dietary protein found in wheat, barley and rye, in genetically predisposed individuals. Rheumatoid arthritis, on the other hand, is an autoimmune disease characterized by chronic inflammation of the synovium of multiple joints. The association of coeliac disease with other autoimmune diseases has been a subject of research and debate. Collin et al studied the associated disorders of 335 coeliac patients and found a significant association with other autoimmune diseases especially with type I diabetes mellitus and Sjogren syndrome [1]. Here we report a case of coeliac disease with rheumatoid arthritis.

## Case Report

**Figure 1:**
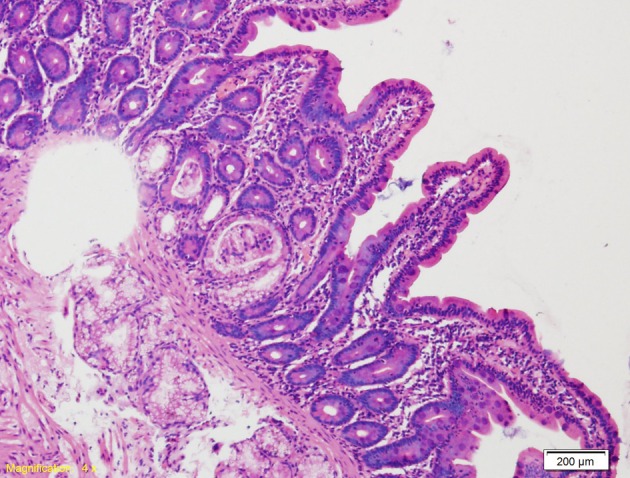
Duodenal mucosa showing infiltration of mononuclear cells with villous atrophy.

A 50-year-old male patient presented with passage of loose stool for 2 months with swelling of both legs for 1 month. There was no history of passage of blood in stool or any history of associated fever. There was no history of any major illness in the past including tuberculosis. On examination patient had pallor with pedal edema. No organomegaly was present. Cardiovascular and respiratory system examination was normal. Blood investigations revealed Hb 9.2 g%, TLC 6,000/cumm, platelets 1.2 lakhs, and ESR 40 mm/h. Liver function test was normal except for a serum albumin 2.1 g/dL. Renal function test was normal. Urine for routine examination was normal. Keeping in mind about patient’s loose stool, loss of weight, hypoalbuminemia and pedal edema, duodenal biopsy was carried out. Biopsy sample from duodenum on histopathological examination revealed infiltration of mononuclear cells with villous atrophy (Fig. 1). Blood for anti-tissue transglutaminase IgA level was 59.4 U/L. Patient was started on a gluten-free diet following which his diarrhea resolved and clinical condition improved. After 3 months on a gluten-free diet, patient’s symptoms improved significantly. However, he swelling involving to started to experience joint pain with swelling involving bilateral wrist, bilateral metacarpophalangeal joints, proximal interphalangeal joints involving both upper limbs and bilateral knee joints. Blood for rheumatoid factor (RF) and C-reactive protein (CRP) were positive with an ESR of 102 mm/h. Blood for anti-CCP level was > 340 U/mL. Keeping in mind patient’s clinical profile and biochemical reports, he was diagnosed to be suffering from rheumatoid arthritis. Patient was started on DMARD monotherapy with methotrexate along with NSAIDS to which the patient responded well. Patient was diagnosed as a case of coeliac disease with rheumatoid arthritis and put on gluten-free diet with DMARDs.

## Discussion

With significant research and insights into the genetic and immunological pathways involved in the pathogenesis of coeliac disease, it is now considered more of a systemic immune-mediated disorder than just a gluten-mediated enteropathy. Coeliac disease shares common pathogenic mechanisms with other autoimmune disorders as evident by its association with many autoimmune disorders. Many studies showed an increase incidence of autoimmune diseases in patients of coeliac disease which include type I diabetes mellitus, autoimmune thyroid diseases and Sjogren’s syndrome [1, 2]. The role of gut-derived antigens in the pathogenesis of many autoimmune diseases is currently a subject of great interest in research. Gut-derived antigens may play an important role as initiators and drivers of inflammation in many autoimmune disorders including rheumatoid arthritis. The manifestations of coeliac disease are a result of interplay between environmental, immunological and genetic factors. Coeliac disease is characterized by an abnormal activation of the immune system in the intestine to an exogenous antigen (gluten) leading to inflammation, tissue destruction and increase in intestinal permeability. In genetically susceptible patients, gluten exposure acts as a driver of autoimmunity and maintains the disease process as evident from the fact that withdrawal of gluten from the diet leads to resolution of the disease. The altered intestinal permeability to gut-derived antigens coupled with a genetic predisposition and altered immunological response may result in a systemic immune response that is directed against sites distant from the gut. Many of the susceptibility genetic loci for coeliac disease are shared with those for rheumatoid arthritis suggesting shared immunological and autoimmune mechanisms [3, 4]. In 1996, Kadioglu and Sheldon in their study suggested that aberrant lymphocytes from the gut mucosal associated lymphoid tissue (MALT) of rheumatoid arthritis patients may migrate pathologically to the synovial fluids [5]. These aberrant circulating lymphocytes originating from the gut and migrating into the joint space may contribute to the pathogenesis of rheumatoid arthritis. There have been reports of coeliac disease associated with rheumatoid arthritis [6]. There are also studies reporting improvement of symptoms of rheumatoid arthritis with dietary modifications suggesting the role of gut-derived antigens as a driver of the inflammatory process in the joints [7, 8]. Podas et al studied the effect of a 2-week elemental diet on 21 rheumatoid arthritis patients and compared them to nine rheumatoid arthritis patients who were given a course of oral prednisolone (15 mg/day). The study reported improvements of subjective clinical parameters in patients with active rheumatoid arthritis which was comparable to improvements seen in patients receiving 15 mg daily dose of prednisolone. In the study Podas et al suggested that rheumatoid arthritis is a disease that starts in the gut [9]. All these studies indicate the important role the gut plays in the pathogenesis of autoimmune disorders. Here we are reporting a case of coeliac disease associated with rheumatoid arthritis. The altered intestinal permeability in coeliac disease exposed the patient to exogenous antigens which could have been the initial trigger and possibly a driver of an autoimmune response in the joints of a genetically susceptible patient. Further research is required to fully understand the role of gut-derived antigens in the pathogenesis of autoimmune diseases.
